# New Publications

**Published:** 1848-02

**Authors:** 


### NEW PUBLICATIONS.

The following new publications were received too late to be noticed in the present number. We shall speak of them in our next:

Practical Physiology; for the use of schools and families. By Edward Jarvis, M. D.

Treatise on Generation; by T. L. G. Bischoff, M. D. etc. Translated from the German by C. R. Gilman, M. D., Professor of Obstetrics, &c., and Theodore Telkampf, M. D., Gebhard Professor, Columbian College.

